# Supplementary material for: Transcriptome Analysis Reveals Vimentin-Induced Disruption of Cell–Cell Associations Augments Breast Cancer Cell Migration
Source: Cells. 2022 Dec 13;11(24):4035. doi: 10.3390/cells11244035 (PMC9776984; doi:10.3390/cells11244035)
Supplement: Supplementary file 1 [file cells-11-04035-s001.zip › DEGs and enrichment analysis.zip!/DEGs and enrichment analysis/WT_VIMvsVector GO enrichment/UP/WT_VIMvsVector_up.BPbar.pdf]

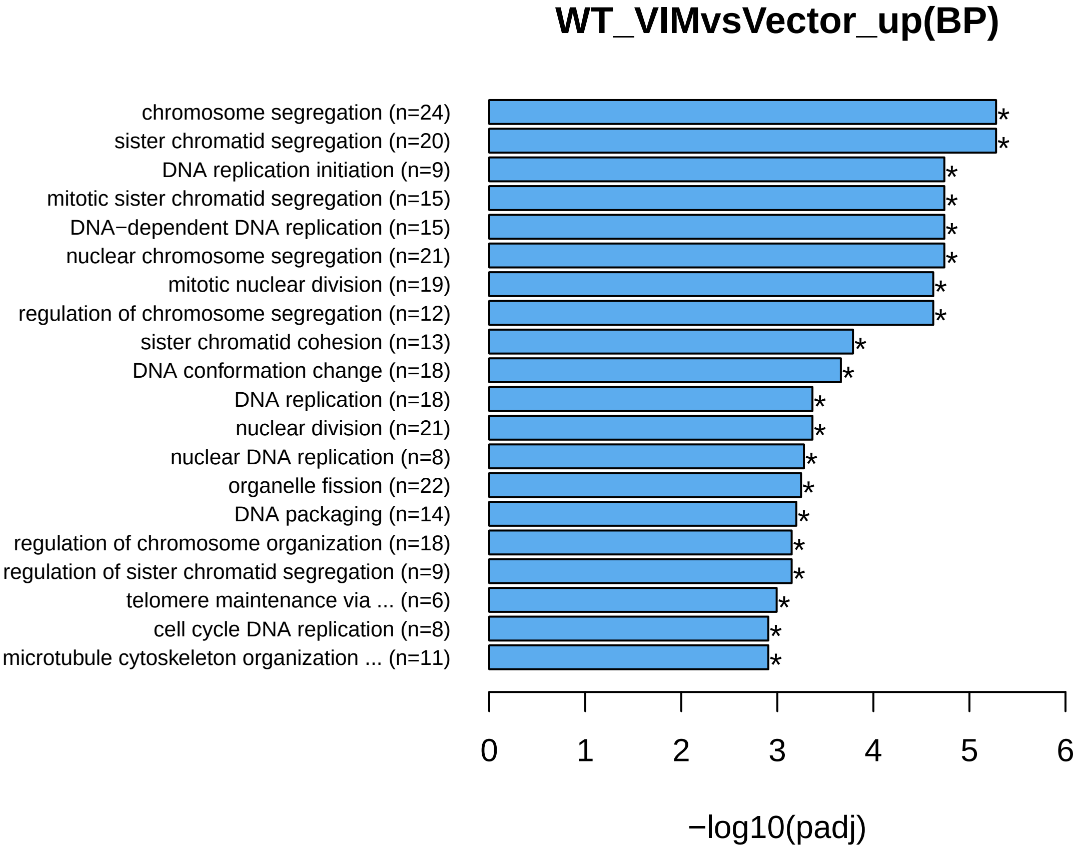
WT_VIMvsVector_up(BP)
chromosome segregation (n=24)
sister chromatid segregation (n=20)
DNA replication initiation (n=9)
mitotic sister chromatid segregation (n=15)
DNA−dependent DNA replication (n=15)
nuclear chromosome segregation (n=21)
mitotic nuclear division (n=19)
regulation of chromosome segregation (n=12)
sister chromatid cohesion (n=13)
DNA conformation change (n=18)
DNA replication (n=18)
nuclear division (n=21)
nuclear DNA replication (n=8)
organelle fission (n=22)
DNA packaging (n=14)
regulation of chromosome organization (n=18)
regulation of sister chromatid segregation (n=9)
telomere maintenance via ... (n=6)
cell cycle DNA replication (n=8)
microtubule cytoskeleton organization ... (n=11)
0
1
2
3
4
5
6
−log10(padj)
